# A biomimetic nanomedicine alleviates liver transplant-related biliary injury by sequentially inhibiting oxidative stress and regulating macrophage polarization via Nrf-2/HO-1 and JNK pathways

**DOI:** 10.1016/j.mtbio.2025.101797

**Published:** 2025-04-23

**Authors:** Tian Dong, Chengcheng Zhang, Zhaoyi Wu, Ling Shuai, Nengsheng Fu, Yujun Zhang, Leida Zhang, Xiang Xiong

**Affiliations:** Key Laboratory of Hepatobiliary and Pancreatic Surgery, Institute of Hepatobiliary Surgery, Southwest Hospital, Third Military Medical University (Army Medical University), Chongqing, 400038, China

**Keywords:** Liver transplantation, Biliary injury, Biomimetic nanoparticles, Nrf-2/HO-1 signaling pathway, JNK signaling pathway

## Abstract

Liver transplantation is an effective method for treating end-stage liver disease. However, 10–20 % of liver transplantation patients develop biliary injury, the main cause of which is ischemia-reperfusion injury (IRI), which consists of oxidative stress injury in the early stage and inflammatory injury in the advanced stage. Biliary injury seriously affects patient outcomes and even leads to mortality, and there are few effective treatments for IRI. Herein, nanoparticles containing quercetin (QR) and rapamycin (RP) coated with poly (lactic-co-glycolic acid) (PLGA) and encapsulated by platelet membrane (PM) were designed to treat IRI in the liver transplantation. The specific binding of ICAM-1 expressed on the PM to integrins (e.g., LFA-1 and Mac-1) in damaged vascular endothelial cells, as well as the interaction between P-selectin on the platelet surface and PSGL-1 on the macrophage surface, allows the accumulation of these biomimetic cell membrane-encapsulated nanoparticles, and subsequently, the delivery of both drugs, to ischemia-reperfusion sites in the liver. The encapsulated QR alleviated oxidative stress injury by activating the Nrf-2/HO-1 signaling pathway in the early stage in model rats with IRI and liver transplantation models. Moreover, RP alleviated inflammatory damage in the advanced stage by suppressing the JNK signaling pathway in M1 macrophages. Thus, these biomimetic nanoparticles that intervene in IRI to alleviate both the early oxidative stress and the advanced inflammatory response constitute a novel delivery system for managing biliary injury after liver transplantation.

## Highlights

• Utilizes platelet membrane targeting to enhance drug accumulation at the site of injury.
• Simultaneous delivery of two drugs, targeting different stages of the injury mechanism, with the synergistic effect of the two drugs being more effective than a single drug.
• Reducing oxidative stress through QR activation of Nrf-2/HO-1 pathway in early stage, and regulating macrophage polarization through RP inhibition of JNK pathway in advanced stage.

## Graphical abstract

A biomimetic nanomedicine that synergistically treats early oxidative stress injury and advanced macrophage polarization via Nrf-2/HO-1 and JNK pathway in a sequential manner was designed to treat biliary injury induced by ischemia‒reperfusion during liver transplantation.

**Figure:**
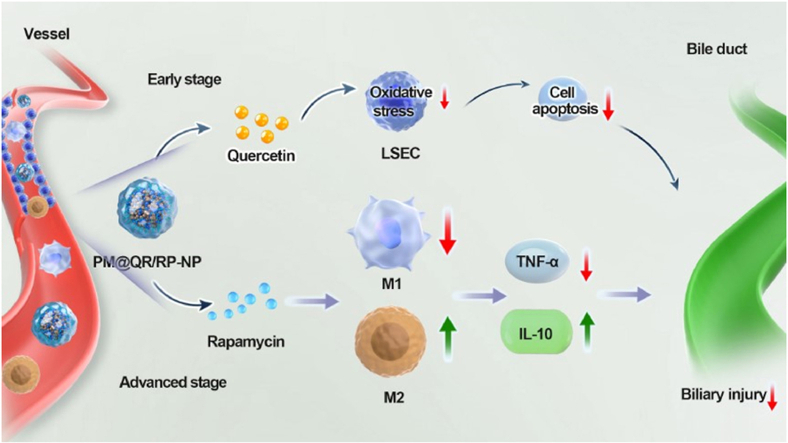

## 1 Introduction

Liver transplantation, the sole effective treatment for end-stage liver disease, has been extensively employed worldwide [1,2]. Nevertheless, 10 %–20 % of liver transplantation patients suffer from severe biliary injury, necessitating retransplantation or causing mortality [3,4]. Targeted drug therapies for biliary injury are still lacking because the exact pathogenesis of biliary injury has not been fully elucidated [5]. However, biliary injury is usually related to hepatic ischemia-reperfusion injury (IRI) that results from the changes in liver tissue metabolism during liver transplantation [6,7].

IRI frequently occurs during surgical procedures and severely restricts the indications for liver resection, the submission of marginal livers for donation, and even the application and therapeutic efficacy of liver transplantation [8,9]. Liver IRI injury has been divided into early and advanced stages, both of which affect the biliary system [[10], [11], [12]]. In the early stage of IRI, oxidative stress injury impairs the function of the mitochondrial respiratory chain complex in liver sinusoidal endothelial cells (LSECs), subsequently generating large amounts of reactive oxygen species (ROS), inducing apoptosis and promoting the overexpression of adhesion factors such as ICAM-1, VCAM-1, and MCP-1 [[13], [14], [15]]. In particular, hypoxia severely damages LSECs, leading to vasoconstriction and luminal narrowing, which reduces the supply of blood and oxygen to cholangiocytes [16,17]. The clinical treatment of oxidative stress depends mainly on antioxidant drugs such as N-acetylcysteine and acetyl-L-carnitine [18,19]. However, the therapeutic effects of these drugs are limited by issues such as low bioavailability and instability. Notably, studies have suggested that nanoparticles may be able to alleviate the vascular damage caused by oxidative stress, providing a new clinical solution [[20], [21], [22]].

During advanced IRI, Kupffer cells, which are specialized macrophages in the liver, recognize the many damage-associated molecular patterns (DAMPs) and high levels of ROS released by LSECs in response to oxidative stress injury [[20], [21], [22]]. This leads to the recruitment of additional Kupffer cells and monocyte-macrophages from the circulatory system and their polarization into M1-type macrophages through the JAK/STAT, PI3K/Akt, and Notch signaling pathways [[23], [24], [25], [26]]. The JNK signaling pathway is highly activated in M1 macrophages, which results in the production of high levels of proinflammatory cytokines, such as TNF-α and IL-12 [12,[27], [28], [29]]. These inflammatory factors further exacerbate the inflammatory response and, together with other factors produced during oxidative stress, initiate a cascade of bile duct cell apoptosis, leading to increased biliary tract damage [[30], [31], [32], [33]]. Therefore, modulating macrophage polarization may be an effective treatment for posttransplant biliary injury.

**Scheme 1:**
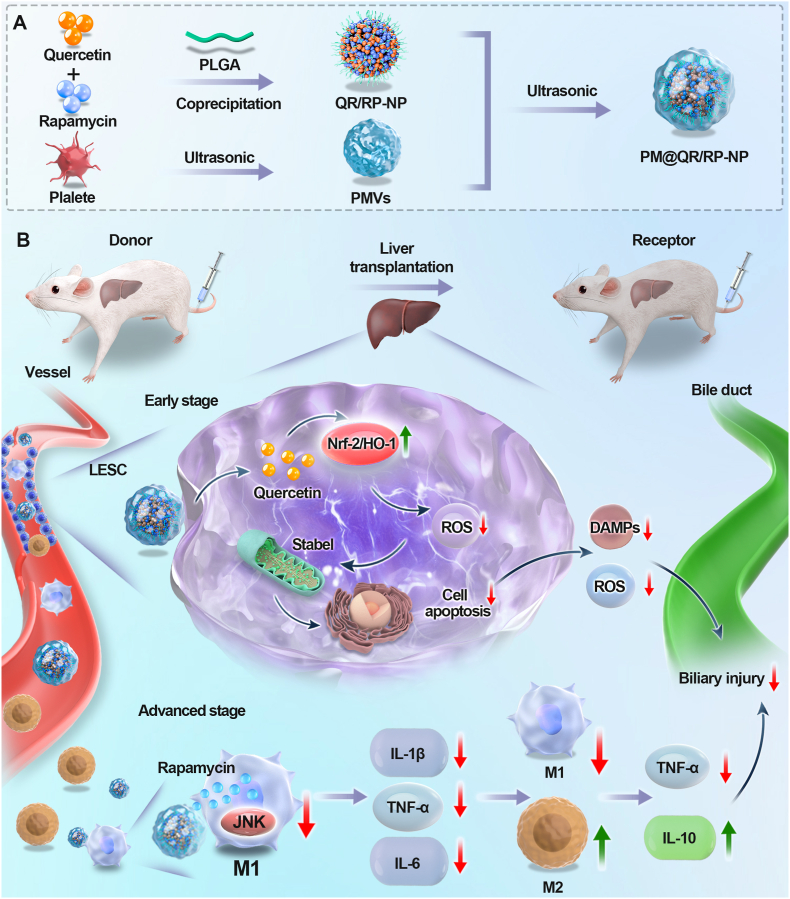
Schematic illustration of the PM@QR/RP-NP preparation (Scheme A). The expression of the Nrf-2/HO-1 signaling pathway is enhanced by using quercetin to clear ROS and maintain mitochondrial stability, thereby alleviating oxidative stress damage to endothelial cells in the early stage of IRI. In the middle and late stages of IRI, rapamycin is used to reduce the JNK signaling pathway in M1 macrophages, alleviating the effects of inflammatory factors on the inflammatory microenvironment and promoting macrophage polarization to M2, thereby solving the long-term inflammatory damage caused by excessive release of inflammatory factors by synergistic treatment to alleviate biliary complications after liver transplantation (Scheme B).

In this study, platelet membrane (PM) -coated biomimetic nanoparticles encapsulating quercetin (QR) and rapamycin (RP) in a poly (lactic-co-glycolic acid) (PLGA) core (PM@QR/RP-NP) were designed to alleviate biliary injury after liver transplantation by addressing both the oxidative stress and immunologic injury that occur during IRI (Scheme 1A). The specific binding of ICAM-1 on PM with the integrins LFA-1 and Mac-1 in damaged vascular endothelial cells and the interaction between P-selectin on the surface of platelets and PSGL-1 on the surface of macrophages allowed the biomimetic nanoparticles to target aggregation at IRI sites after liver transplantation [[34], [35], [36]]. Delivered QR activated the Nrf-2/HO-1 signaling pathway in LSECs to scavenge ROS and maintain mitochondrial stability, thereby attenuating oxidative stress injury in LSECs in early IRI, maintaining normal function of periportal vasculature, and securing blood and oxygen supply to cholangiocytes. In the advanced stage of IRI, RP released into macrophages inhibited the JNK signaling pathway in M1 macrophages, which attenuated the role of pro-inflammatory factors in the inflammatory microenvironment, while tending to polarize M2 macrophages, increase the number of M2-type macrophages, and increase the secretion of anti-inflammatory factors, which maintained an anti-inflammatory microenvironment and solved the long-term damage to cholangiocyte caused by the over-release of inflammatory factors (Scheme 1B). Thus, the full synergistic potential of these cell-targeting biomimetic nanoparticles is exploited by suppressing oxidative stress in the early stage and resisting M1 macrophage-mediated inflammation in the advanced stage, offering a possible treatment for biliary injury after liver transplantation.

## 2 Methods

### 2.1 Enrichment analysis

GO enrichment analysis using data from one published article [37],the findAlMarkers function with the default settings was employed to identify the differentially expressed genes (DEGs) in each cluster. DEGs were classified as upregulated or downregulated when their average log2FC values were greater than or less than 0.25, respectively. Subsequently, enrichment analyses of the upregulated and downregulated DEGs were conducted using clusterProfiler 4.2.2.

### 2.2 Preparation of PLGA nanoparticles coloaded with QR and RP

QR/RP-NP were fabricated via a coprecipitation-solvent evaporation method as follows. First, appropriate amounts of PLGA, QR, and RP (PLGA: QR:RP of 20:2:1 w/w/w) were dissolved in a suitable amount of acetone, and this mixture was slowly added to a 2 % (w/w) polyvinyl alcohol (PVA) solution. The resulting mixture was continuously and rapidly stirred at room temperature overnight. The PLGA nanoparticles encapsulating QR and RP were collected by ultrafiltration.

### 2.3 Extraction of the PM

Blood from male SD rats was collected in an EDTA tube and centrifuged at 200×*g* for 20 min at room temperature, after which the supernatant containing platelet-rich plasma (PRP) was collected. Then, a 5 mM prostaglandin E1 (PGE1) solution in phosphate-buffered saline (PBS) was added to the purified PRP, and the platelets were separated by centrifugation at 1800×*g* for 20 min at room temperature. After the supernatant was removed, the platelets in the pellet were resuspended in PBS containing 5 mM PGE1, were subjected to repeated freeze-thaw cycles, centrifugation at 8000×*g* for 15 min, and sonication for 2 min in a bath sonicator. Finally, the PM was aliquoted into 1 mL samples and stored at −80 °C until use.

### 2.4 Preparation of the drug-loaded biomimetic nanoparticles

The surface of the PM@QR/RP-NP were modified with the collected PM via ultrasonic treatment to obtain PM@QR/RP-NP as follows. First, an appropriate amount of PM@QR/RP-NP dispersed in 2 % (w/w) PVA solution was mixed with an equal weight of cell membrane vesicle dispersion solution. The mixture was subjected to ice-bath ultrasonic treatment (100 W) cycles with 2 min on and 2 min off, which was repeated until the cell membranes covered the QR/RP-NP surface. The prepared PM@QR/RP-NP were collected by ultrafiltration and stored at −80 °C until use.

### 2.5 Characterization of the drug-loaded nanoparticles

Particle size and zeta potential: The hydrodynamic particle sizes and zeta potentials of the PMVs, QR/RP-NP, and PM@QR/RP-NP dispersed in PBS (1 mg mL^−1^) were measured via DLS at 25 °C.

Morphological characterization: One drop of each the PMVs, QR/RP-NP, and PM@QR/RP-NP dispersion (1 mg mL^−1^) was added onto the carbon film side of a copper grid and allowed to stand for deposition. The samples were subsequently stained with a 2 % (w/w) phosphotungstic acid solution for 1 min and air-dried. The morphological structures of the various nanoparticles were observed and photographed by TEM.

Detection of the cell membrane proteins on the PM@QR/RP-NP: Successful modification of the PM@QR/RP-NP with the cell membrane and the integrity of the cell membrane proteins during preparation were verified via SDS-PAGE. The gel was prepared following standard SDS-PAGE procedure and stained with Coomassie Brilliant Blue staining solution for 2 h after sample separation. After the staining solution was removed, the protein band distributions of in the different samples was observed. PM markers were detected by Western blotting.

### 2.6 Evaluation of nanoparticle drug loading and drug release behavior

Drug loading capacity (LC) and drug loading efficiency (LE): After establishing ultraviolet absorption standard curves of QR and RP, the drug concentrations in the PM@QR/RP-NP and QR/RP-NP were determined by high-performance liquid chromatography. Then, the LCs and LEs of QR and RP in the various nanoparticles were calculated.

$$\text{LC}=\left(\;\frac{\text{Mass}\;\text{of}\;\text{drug}\;\text{encapsulated}\;\text{in}\;\text{PLGA}\;\text{nanoparticles}}{\text{Total}\;\text{mass}\;\text{of}\;\text{drug}\;\text{in}\;\text{PLGA}\;\text{nanoparticles}}\right)\times 100\%$$

$$\text{LE}=\left(\;\frac{\text{Amount}\;\text{of}\;\text{drug}\;\text{encapsulated}\;\text{in}\;\text{PLGA}\;\text{nanoparticles}}{\text{Total}\;\text{amount}\;\text{of}\;\text{drug}\;\text{in}\;\text{PLGA}\;\text{nanoparticles}}\right)\times 100\%$$

Drug release: QR-NP and PM@QR-NP containing 2 mg of QR were dispersed in 15 mL of PBS, and each dispersion was equally divided in half and placed in dialysis bags. The dialysis bags were placed in centrifuge tubes containing PBS (pH = 7.4) or Tris-HCl buffer (pH = 5.0) and shaken at 37 °C. At predetermined time points, 1 mL of buffer was removed from the centrifuge tubes, and an equal volume of buffer was added back to the centrifuge tube. The same procedures were performed for the PM@RP-NP and RP-NP. On the basis of the standard curves of QR or RP, the *in vitro* drug release from the QR-NP, PM@QR-NP, RP-NP, and PM@RP-NP at pH 5.0 and pH 7.4 were calculated.

### 2.7 Intracellular localization of the nanoparticles

ECs were seeded in confocal culture dishes at a density of 4 × 10^5^ cells/well and cultured for 24 h for adherence. PM@NR-NP were added such that the final concentration of Nile red in the confocal culture dish was 10 μg mL-1, and the cells were incubated for an additional 1, 3, or 6 h. The ECs were subsequently stained with lysosome fluorescent probes (green) and DAPI, and the intracellular localization of the nanoparticles was observed using a laser confocal microscopy.

### 2.8 Alleviation of oxidative stress *in vitro*

ECs were seeded in 6-well plates at a concentration of 5 × 10^5^ cells/well and cultured for 24 h for adherence. Next, an appropriate amount of H_2_O_2_ was added to the experimental group media to reach a final concentration of 1 mM. Then, different concentrations of QR/RP, QR-NP, QR/RP-NP, or PM@QR/RP-NP were added for 24 h of incubation, after which the cells were stained with fluorescent ROS probes, JC-1, or TUNEL staining reagents. The ECs were finally observed by fluorescence microscopy to evaluate the alleviation of antioxidant stress by the different QR preparations.

### 2.9 Regulation of macrophage polarization *in vitro*

Macrophages were seeded in 6-well plates at a density of 5 × 10^5^ cells/well and cultured for 24 h. Then, appropriate amounts of LPS and IFN-γ were added to the medium, and the cells were incubated for an additional 12 h. Then, QR/RP, RP-NP, QR/RP-NP, or PM@QR/RP-NP were added, and the cells were further incubated for 24 h. Immunofluorescence staining of CD86 and CD206 was performed on the cell climbing slices, the proteins were extracted for Western blotting, and the culture media supernatants were collected. The concentrations of the cytokines TNF-α and IL-10 in the culture media from each group were detected using ELISA kits to evaluate the regulation of macrophage polarization.

### 2.10 Rat IRI model

Rats were anesthetized by intraperitoneal injection of 1 % pentobarbital sodium, and the arterial/portal blood flow of the left lobe of the liver was occluded for 60 min using noninvasive clips. The clips were subsequently removed to achieve reperfusion. The rats in the sham operation group underwent the same procedure, except that the arterial/portal blood flow was not occluded.

### 2.11 Rat liver transplantation mode

The livers of the donor rats were subjected to 30 min of warm ischemia. After the recipient rat was placed under anesthesia, the abdominal cavity was exposed by making a midline incision in the upper abdomen, and the recipient liver was gradually separated and removed. Next, the donor liver was placed in the same position as the recipient liver, and the superior and inferior vena cava and the portal vein were anastomosed. Finally, the donor bile duct was inserted into the recipient bile duct cannula, and the abdominal wall was sutured layer by layer. Tissue samples were collected on the 1st and 7th day after transplantation, and transplant injury was scored on the basis of parameters such as tissue morphology, pathological scoring, liver function, immunofluorescence staining, Western blotting, and inflammatory factor levels.

### 2.12 Liver-targeting ability of the nanoparticles

A liver ischemia/reperfusion model was established in SD rats by intravenously injecting the same dose (10 mg kg^−1^) of saline, NR, NR-NP, or PM@NR-NP. At predetermined time points after the injection, rats were anesthetized, and fluorescence distributions were detected *in vivo* using an *in vivo* imaging system, and the same mass of visceral tissues was weighed and centrifuged into the supernatant for measurement of the tissue fluorescence intensity. The fluorescence intensity of the tissue was measured.

$$\%\;\text{ID}=\left(\;\frac{\text{dose}\;\text{of}\;\text{tissue}\;\text{sample}e}{\text{injected}\;\text{dose}}\right)\times 100\%$$

$$\%\;\text{ID}/g=\frac{\;\%\;\text{ID}}{\;\text{weight}\;\text{of}\;\text{tissue}\;\left(g\right)}$$

### 2.13 Evaluation of drug administration effect

IRI rats were given the drug intravenously in the tail at 24 h prior to liver IRI, and tissue samples were collected 12 h after postoperative administration. Donor rats for liver transplantation were given the drug in the tail vein 24 h before liver transplantation and recipients were given the drug every other day after transplantation. Tissue samples were collected on the first and seventh day after transplantation, respectively. The therapeutic effect was evaluated based on parameters such as histomorphology, pathology scores and liver function, immunofluorescence staining of tissue samples, protein blotting and inflammatory factor levels.

### 2.14 Western blotting

Total protein was extracted from treated cells or tumor tissues in RIPA lysis buffer and separated by 8 % or 10 % sodium dodecyl sulfate-polyacrylamide gel electrophoresis (SDS-PAGE) (Beyotime, China) before being transferred onto PVDF membranes (Bio-Rad Laboratories, USA). The membranes were blocked in 5 % skim milk for 1 h at room temperature and then incubated with primary antibody overnight at 4 °C. Subsequently, the membranes were incubated with secondary antibody for 1 h at room temperature. The immunoreactive bands were detected using Clarity TM Western ECL substrate (Bio-Rad, USA) and a Bio-Rad GelDoc system (Bio-Rad, USA).

### 2.15 H&E and immunofluorescence staining

Dissected liver and bile duct tissues were fixed overnight in 10 % paraformaldehyde, then embedded in paraffin and serially sectioned to 3–5 mm thickness. For HE staining, tissue sections were sequentially deparaffinized, rehydrated (in a graded ethanol series), and stained with hematoxylin by the traditional method, followed by eosin staining for approximately 20 s. The tissue was then dehydrated and covered with neutral gum. Images were captured using a light microscope. Suzuki scoring, BDDS and BDISS scoring of H&E staining were performed by different specialized pathologists who were not aware of the rat subgroups. For immunofluorescence staining, citrate buffer (pH 6.0; 10 mmol/L citric acid, 0.05 % Tween 20) was used, followed by heating in a high-pressure boiler for antigen repair. Sections were then incubated with methanol containing 3 % hydrogen peroxide for 15 min to inhibit endogenous peroxidase activity, and then the sections were blocked with TBS containing 3 % goat serum. Subsequently, the sections were incubated overnight at 4 °C with antibody CK7 and incubated with the corresponding secondary antibody for 60 min at room temperature to repeat the antigen repair blocking and blocking process. Sections were incubated overnight at 4 °C with antibodies F4/80, CD86, or CD206, incubated with the corresponding secondary antibodies for 60 min at room temperature, and blocked by labeling the nuclei with DAPI. Images were acquired using a fluorescence microscope (BX53, Olympus).

## 3 Results and discussion

### 3.1 Clinical features of the ECs and macrophages around bile ducts after liver transplantation

**Fig. 1:**
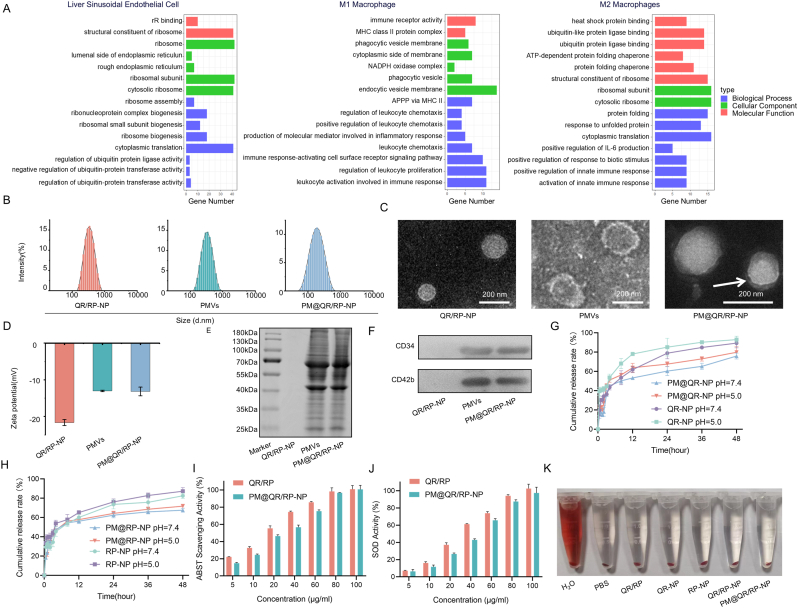
Clinical features and characteristics of biliary injury after liver transplantation and characterization of PM@QR/RP-NP. A) GO enrichment analysis of differentially expressed genes in non-anastomotic biliary stricture after liver transplantation compared with patients who did not develop non-anastomotic biliary strictures. B) Size distribution analysis of QR/RP-NP, PMVs, and PM@QR/RP-NP. C) TEM microscopic photos showing the morphology of QR/RP-NP, PMVs, and PM@QR/RP-NP (Scale bar = 200 nm). D) ζ-potential of QR/RP-NP, PMVs, and PM@QR/RP-NP. E) Coomassie brilliant blue stained SDS-PAGE gel images of QR/RP-NP, PMVs, and QR@RP/RP-NP. F) Western blot images of CD34 and CD42b in PM@QR/RP-NP, QR/RP-NP, and PMVs. G) Release curves of QR from QR-NP and PM@QR-NP in buffers of pH 7.4 and pH 5.0. H) Release curves of RP from RP-NP and PM@RP-NP in buffers of pH 7.4 and pH 5.0. I) ABST clearance activity of QR/RP and PM@QR/RP-NP. J) SOD activity of QR/RP and PM@QR/RP-NP. K) Hemolysis test results of QR/RP, QR-NP, RP-NP, QR/RP-NP, and PM@QR/RP-NP. Data are presented as mean ± SEM. (For interpretation of the references to color in this figure legend, the reader is referred to the Web version of this article.)

To investigate the relationships among between biliary injury and the peripheral tissues after liver transplantation, gene enrichment analyses were performed by assessing the differences between patients who developed non-anastomotic biliary strictures after liver transplantation and those who did not. Gene enrichment analysis (Fig. 1A) revealed that aerobic respiration was significantly impaired in the perivascular ECs of the extrahepatic bile duct, the expression of normal respiratory signaling pathways was reduced, and the expression of inflammation-related pathways (e.g., immune receptor activity and cytokine secretion) was increased in M1 macrophages. Compared with those who did not develop non-anastomotic biliary strictures, patients who did develop biliary injury had reduced M2 macrophage activity and suppressed anti-inflammatory pathways. These results suggested that the postoperative oxidative stress and macrophage polarization caused by IRI may exacerbate biliary injury after liver transplantation.

On the basis of the GO analysis results, PM@QR/RP-NP were designed (Scheme 1A) to alleviate liver transplant-related biliary injury. In previous experimental studies, quercetin mainly protects endothelial cells to attenuate oxidative stress injury by activating the Nrf-2/HO-1 pathway[[38], [39], [40]]. Rapamycin regulates macrophage polarization status by inhibiting the mTOR signaling pathway and promotes macrophage polarization toward the M2-type, thus suppressing inflammatory responses[[41], [42], [43]]. To address the shortcomings of quercetin's low bioavailability and rapamycin's toxic side effects, biomimetic nanoparticle-encapsulated drugs were designed to improve the pharmacokinetic deficiencies of the two and to take advantage of targeted drug delivery and enhanced efficacy.

### 3.2 Characterization of the PM@QR/RP-NP

Physicochemical characterization of PM@QR/RP was examined. Dynamic light scattering (DLS) analysis showed that the size of the PM@QR/RP-NP was 126.5 ± 34.98 nm (Fig. 1B), and that passive targeting enhanced nanocarrier accumulation in the liver [44]. Transmission electron microscopy (TEM) observations (Fig. 1C) showed that the QR/RP-NP were spherical, whereas the assembled PM@QR/RP-NP, indicated by the arrow, had a unique core-shell structure, suggesting that the PM coated the nanoparticle surface. Furthermore, the zeta potentials (Fig. 1D) of the QR/RP-NP, platelet membrane vesicles (PMVs), and PM@QR/RP-NP were −22.63 ± 0.81 mV, −13 ± 0.2 mV, and −13.16 ± 1.17 mV, respectively, and the similar potentials of the PMVs and PM@QR/RP-NP further confirmed that the PM had been successfully coated on the surface of the nanoparticles. SDS-PAGE (Fig. 1E) revealed that the protein bands of the PMVs and PM@QR/RP-NP were not significantly different, whereas the no protein bands were observed for the QR/RP-NP sample. Furthermore, the Western blot (WB) experiments (Fig. 1F) showed that the PM@QR/RP-NP and PMVs expressed CD34 and CD42b, two signature proteins of the PM, which indicated that the synthesized PM@QR/RP-NP might retain the biological function of platelets.

Subsequently, buffer solutions of different pH values were used to simulate different pathways of biomimetic nanoparticle release in the human body. The LC and LE of QR in PM@QR/RP-NP were 3.14 ± 0.65 % and 61.38 ± 4.64 %, respectively, while that of RP were 1.02 ± 0.45 %and 43.55 ± 6.21 %, respectively (Table S1). The curves of drug release from the QR-NP and PM@QR-NP (Fig. 1G) revealed that after 48 h at pH 7.4, the cumulative rates of QR release from the QR-NPs and PM@QR-NP were 89.38 % and 75.98 %, respectively. Moreover, the cumulative release percentages of QR from the QR-NP and PM@QR-NP in the pH 5.0 buffer solution were 93.02 % and 79.91 %, respectively. These results indicated that the PM@QR/RP-NP could release QR more effectively in the lysosomes of cells (pH 5.0) than in the circulation (pH = 7.4). A possible reason for this result is that an acidic environment may affect the structure of the PM, allowing the drug to rapidly diffuse out of the nanoparticle. Furthermore, similar conclusions were drawn from the release curves of RP from the RP-NP and PM@RP-NP. The cumulative release rates of RP from the RP-NP and PM@RP-NP were 82.55 % and 67.58 %, respectively, at pH 7.4, and in pH 5.0 buffer (Fig. 1H), these values were 87.31 % and 71.75 %, respectively.

After the physicochemical properties of the PM@QR/RP-NP were assessed, the ability of the nanoparticles to scavenge free radicals was evaluated. The results of the ABTS test (Fig. 1I) revealed that the PM@QR-NP containing 10 μg mL^−1^, 20 μg mL^−1^ and 40 μg mL^−1^ QR could clear 24.54 %, 46.41 %, and 56.69 % of the free radicals, respectively, indicating the scavenging ability of the PM@QR/RP-NP increased along with QR concentration. The results of the SOD activity experiments confirmed that the PM@QR/RP-NP could effectively clear ROS. Furthermore, the hemolysis experiment results (Fig. 1K) showed that the QR-NP, RP-NP, QR/RP-NP, and PM@QR/RP-NP did not cause hemolysis, thus proving that the biomimetic nanomedicine could be administered via the circulatory system.

### 3.3 Antioxidant stress resistance of the PM@QR/RP-NP *in vitro*

**Fig. 2:**
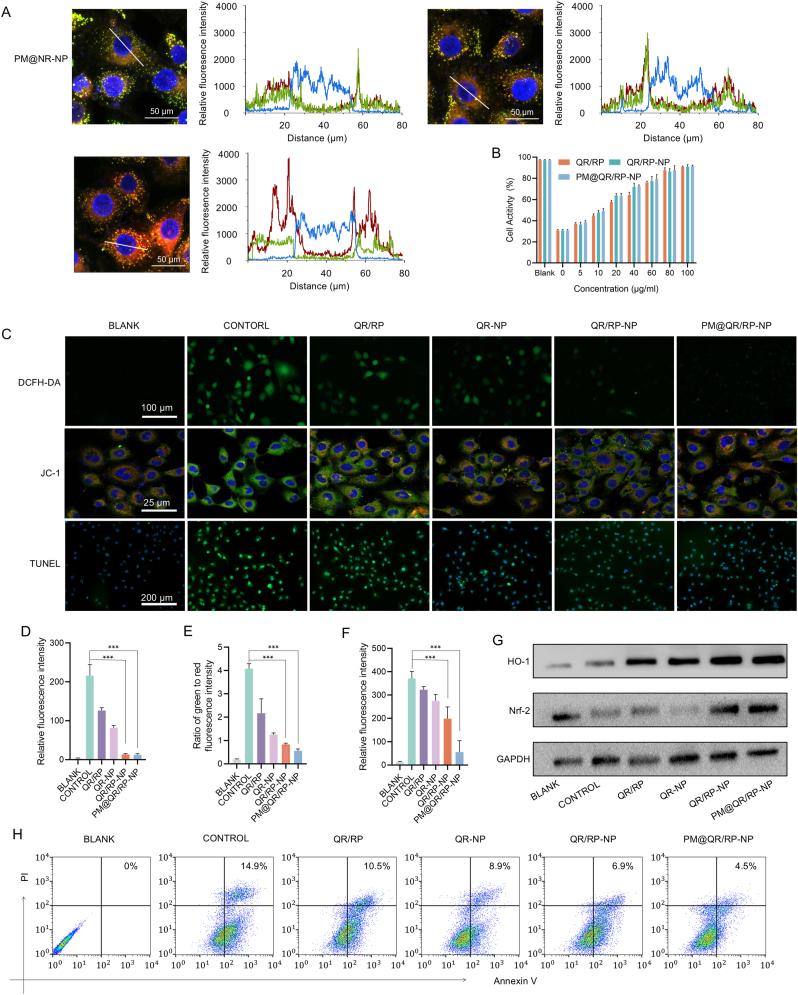
Cellular uptake and antioxidant stress ability of PM@QR/RP-NP. A) Intracellular colocalization of the PM@NR-NP (Nile red is red, lysosomes are green, nuclei are blue) (Scale bar = 50 μm). B) Effects of nanoparticle on ECs viability evaluated by CCK-8 assay (n = 3). C) Fluorescence figures of ECs stained with DCFH-DA (green) (Scale bar = 100 μm), JC-1 (the J-aggregates is red, JC-1 monomer is green) (Scale bar = 25 μm), and TUNEL (green) (Scale bar = 200 μm). Quantitative analysis of relative intensity fluorescence of (D) DCFH-DA, (E) JC-1, and (F) TUNEL in Fig. 2C. H) Flow cytometry analysis of ECs after various treatments. I) Western blot analysis showing the expression of HO-1, Nrf-2 in ECs after various treatments. Data presented as mean ± SEM. ∗p < 0.05, ∗∗p < 0.01, ∗∗∗p < 0.001. (For interpretation of the references to color in this figure legend, the reader is referred to the Web version of this article.)

In this study, the antioxidant capacity of the PM@QR/RP-NP was assessed *in vitro* in Human umbilical vein endothelial cells (ECs). Biomimetic nanoparticles labeled with Nile red (PM@NR-NP) were coincubated with H_2_O_2_-stimulated ECs and then observed by confocal laser scanning microscopy (Fig. 2A). The green fluorescence signal of the lysosomes overlapped with the red fluorescence signal of the nanoparticles, producing a yellow color. Compared with that of the 1-h group, the red fluorescence intensity of the PM@NR-NP 6-h group was greater, indicating that the PM@NR-NP were extensively taken up by ECs and distributed mainly in the lysosomes. A CCK-8 assay was then used to evaluate the cytotoxicity of the PM@QR/RP-NP (Fig. S1), and EC viability remained greater than 80 % after treatment with 0–100 μg mL^−1^ QR/RP-NP or PM@QR/RP-NP, indicating that the PM@QR/RP-NP were not obviously cytotoxic and were safe for subsequent experiments. Furthermore, the viability of ECs exposed to an overload of H_2_O_2_ after 24 h of pretreatment with different concentrations of nanoparticles was further analyzed by a CCK-8 assay (Fig. 2B). The survival rate of the control ECs was 30.86 %, whereas the survival rate of ECs pretreated with 40 μg mL^−1^ PM@QR/RP-NP showed an approximately 40 % recovery compared with the control group, indicating the significant recovery of cell viability. These data suggested that the biomimetic nanoparticles could significantly increase the survival of H_2_O_2_-stimulated cells, suggesting their potential application in the treatment of IRI. Therefore, PM@QR/RP-NP with this concentration of QR were applied in subsequent experiments to ensure the optimal effect.

Furthermore, DCFH-DA staining was used to assess the intracellular ROS content after different treatments to gain insight into the ability of the PM@QR/RP-NP to scavenge ROS (Fig. 2C and D), the staining results showed that H_2_O_2_ stimulation apparently induced ROS overload in ECs, as evidenced by the high intensity of green fluorescence. Compared with that in H_2_O_2_-stimulated ECs, the relative fluorescence intensity in the QR-NP group decreased to 37.96 %, suggesting significant ROS inhibition. Notably, the relative fluorescence intensity of the QR/RP-NP group decreased to 6.18 %, indicating stronger ROS inhibition than that of the single agent group, which may be due to the synergistic ROS clearance ability of the two drugs. These results demonstrate the good antioxidant capacity of the QR/RP-NP.

Oxidative stress can lead to the destruction of mitochondrial morphology and structure, which can subsequently affect cell apoptosis. JC-1 staining can detect changes in the mitochondrial membrane potential, with red indicating a normal mitochondrial potential and green indicating a decreased mitochondrial membrane potential associated with mitochondrial dysfunction. As shown in Fig. 2C and E, the ratio of green to red fluorescence in H_2_O_2_-stimulated ECs was 4.08, indicating that mitochondrial function was greatly affected. However, after treatment with the QR/RP-NP and PM@QR/RP-NP, the green to red fluorescence ratio in H_2_O_2_-stimulated ECs decreased to 0.84 and 0.55, respectively, and were lower than the value of 1.26 in the QR-NP group. Treatment with the PM@QR/RP-NP led to the lowest green to red fluorescence ratio, which may be due to nanoparticle endocytosis by ECs mediated by the PM.

Because PM@QR/RP-NP strongly reduce oxidative stress, we further explored whether they could attenuate apoptosis triggered by oxidative stress. TUNEL staining was used to detect apoptotic cells, which presented an intense green fluorescence signal. As shown in Fig. 2C and F, H_2_O_2_-stimulated ECs presented a strong green fluorescence signal and the green fluorescence intensity of the PM@QR/RP-NP group was 27.8 % that of the QR/RP-NP group, indicating that nanoparticle treatment effectively reduced the degree of apoptosis caused by excessive oxidative stress. This conclusion was supported by the results of the cellular flow cytometry experiments (Fig. 2G), where 14.9 % of the cells underwent apoptosis after H_2_O_2_ stimulation. Furthermore, the percentage of apoptotic cells after treatment with PM@QR/RP-NP (4.5 %) was half that after treatment with the QR-NP (8.9 %).

Nrf-2 is an important transcription factor involved in the antioxidant response [45], and HO-1 is a key protein downstream of Nrf-2 [46]. The WB results (Fig. 2H) showed that the expression levels of Nrf-2 and HO-1 increased significantly after treatment with PM@QR/RP-NP. These findings suggested that encapsulating the drug in the PM may enhance the biocompatibility of the drug, thereby increasing the expression of Nrf-2 and its downstream target protein HO-1. This leaded to the effective elimination of the large amount of ROS generated during IRI and a decreased risk of ECs apoptosis. These findings suggested that PM@QR/RP-NP protect ECs in the early stage of IRI by reducing the damage caused by ROS.

### 3.4 The PM@QR/RP-NP regulate macrophage polarization and inhibit inflammation *in vitro*

**Fig. 3:**
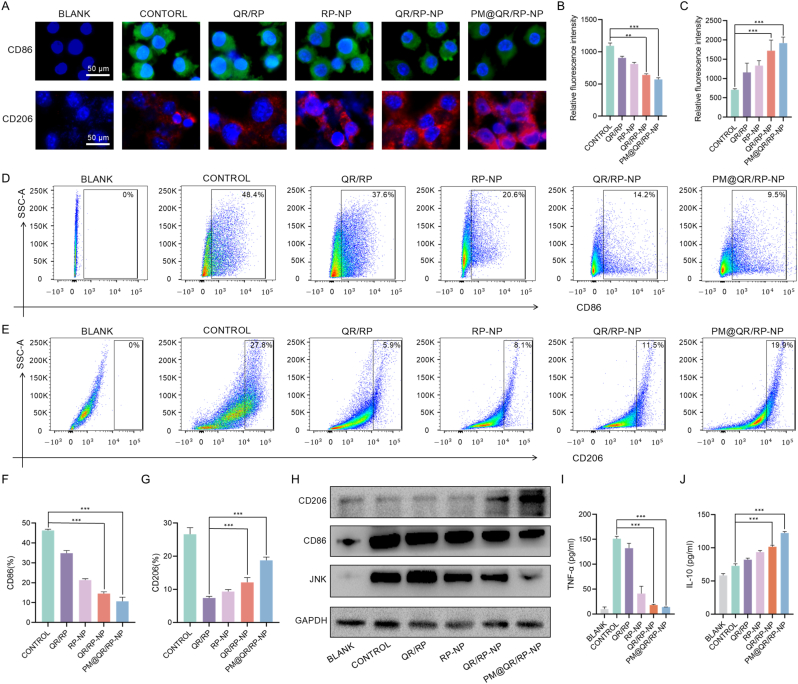
Regulate macrophage polarization and inhibit inflammation of PM@QR/RP-NP *in vitro*. A) Fluorescence images of CD86 (green), CD206 (red), and DAPI (blue) labeled RAW264.7 cells after different treatments. (Scale bar = 50 μm) Quantitative immunofluorescence staining analysis of (B) CD86 and (C) CD206 expression in Fig. 3A. Flow cytometry analysis of (D) CD86 and (E) CD206 positive RAW264.7 cells after different treatments. Quantitative analysis of (F) CD86 and (G) CD206 positive macrophages in flow cytometry analysis. H) Western blot analysis of CD86, CD206, and JNK expression in RAW264.7 cells after different treatments. ELISA analysis of (I) TNF-α and (J) IL-10 expression in RAW264.7 cells after different treatments (n = 3). Data are presented as mean ± SEM. ∗p < 0.05, ∗∗p < 0.01, ∗∗∗p < 0.001. (For interpretation of the references to color in this figure legend, the reader is referred to the Web version of this article.)

IRI involves macrophage polarization in addition to oxidative stress-induced damage in LSECs. In the advanced stage of IRI, the oxidative stress-induced damage to LSECs results in the recruitment of macrophages and promotes their polarization into the M1 type [47,48]. The flow cytometry experiments in this study supported this phenomenon, hydrogen peroxide stimulates ECs to polarize toward M1 macrophages (Fig. S2), but to maintain stability during the experiment, lipopolysaccharide (LPS) and IFN-γ were used to promote macrophage polarization in subsequent experiments. In the advanced stage of IRI, M1 macrophages release proinflammatory cytokines, exacerbating the proinflammatory microenvironment and further promoting the M1 polarization of macrophages and damage to cholangiocytes. To verify that the PM@QR/RP-NP could regulate macrophage polarization, RAW264.7 cells were subjected to different treatments for subsequent immunofluorescence staining. The immunofluorescence images and relative immunofluorescence intensity ratios (Fig. 3A–C) showed that the expression of the M1 macrophage marker CD86 and the M2 macrophage marker CD206 in the blank group were low. In the control group treated with LPS and IFN-γ, the green fluorescence intensity of CD86 was significantly increased, whereas the red fluorescence intensity of CD206 was decreased. Compared with that of the RP-NP group, the green fluorescence intensity of CD86 in the QR/RP-NP group decreased to 79 %, whereas the red fluorescence signal of CD206 increased to 128.96 %, indicating that the QR/RP-NP could better regulate macrophage polarization than the single drugs alone due to the synergistic effect of both drugs. Flow cytometry and quantitative analysis (Fig. 3D–G) revealed that the number of M1 macrophages in the PM@QR/RP-NP group was reduced to approximately 73.16 % of that in the QR/RP-NP group, whereas the number of CD206 macrophages was increased to approximately 154.74 %, indicating that the PM@QR/RP-NP could regulate the polarization of macrophages from the M1 type to the M2 type. The WB results (Fig. 3H) further confirmed the ability of the PM@QR/RP-NP to polarize macrophages. After treatment with PM@QR/RP-NP, the protein expression of CD86 was significantly reduced, whereas the protein expression of CD206 increased. This may affect the activation of the JNK inflammatory pathway, thereby modifying the inflammatory microenvironment. In addition, enzyme-linked immunosorbent assays (ELISA) (Fig. 3I and J) confirmed the expression levels of the inflammatory factor TNF-α and the anti-inflammatory factor IL-10 after PM@QR/RP-NP treatment. RAW264.7 cells treated with LPS and IFN-γ expressed 151.11 pg mL^−1^ TNF-α and 72.90 pg mL^−1^ IL-10. Compared with the control group, the PM@QR/RP-NPs relieved inflammation to a certain extent by decreasing the expression of TNF-α to 14.01 pg mL^−1^ and increasing the expression of IL-10 to 122.39 pg mL^−1^. Therefore, treatment with PM@QR/RP-NP effectively reduced the secretion of inflammatory factors and increased the quantity of anti-inflammatory factors, thus modifying the inflammatory microenvironment.

Because PM@QR/RP-NP can regulate macrophage polarization and alleviate inflammation, the ability of the PM@QR/RP-NP to reduce damage to biliary epithelial cells caused by polarized macrophages *in vitro* was investigated. Extrahepatic bile duct epithelial cells as cholangiocytes were cocultured with treated macrophages, and the CCK-8 assays (Fig. S3) revealed that PM@QR/RP-NP could enhance the activity of cholangiocytes cocultured with macrophages. The TUNEL staining results (Fig. S4) also revealed that the green fluorescence of the control cell nuclei was greater, whereas the green fluorescence signal in the nuclei of cells treated with PM@QR/RP-NP was significantly lower, indicating that these nanoparticles could protect cholangiocytes by regulating macrophage polarization and alleviating the inflammatory response *in vitro*. In summary, these results suggested that PM@QR/RP-NP inhibit M1 macrophage polarization, thereby reducing inflammatory factor release and regulating the inflammatory microenvironment to mitigate the inflammatory response and reduce cholangiocyte damage.

### 3.5 Distribution and biocompatibility of the PM@QR/RP-NP *in vivo*

**Fig. 4:**
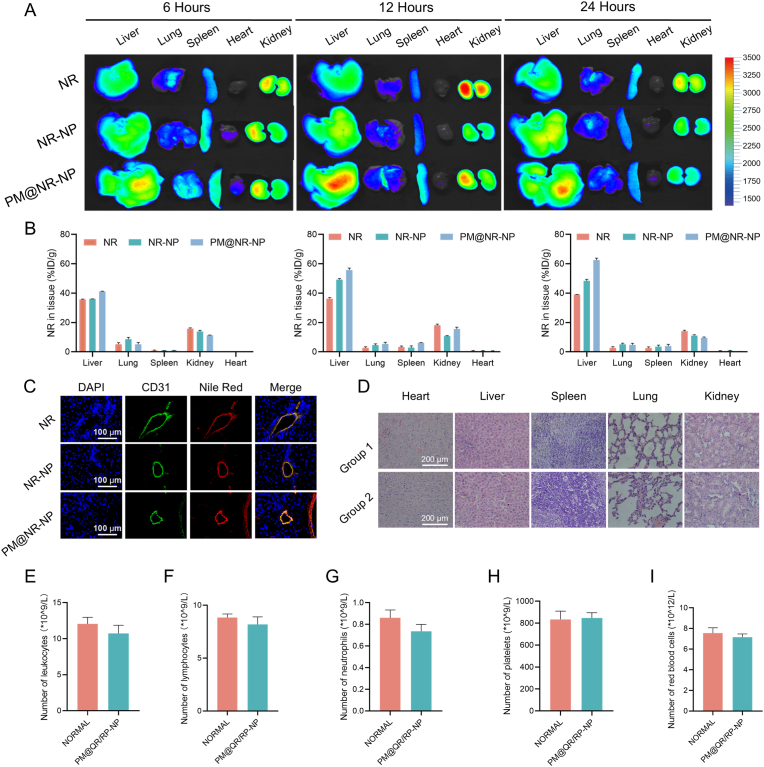
Distribution and biocompatibility of the PM@QR/RP-NP *in vivo* A) *Ex vivo* imaging of the main organs from rats receiving liver transplantation at 6, 12 and 24 h after intravenous injection of NR, NR-NP and PM@NR-NP. B) Quantification of the fluorescence intensities of the organs in Fig. 4A. C) Immunofluorescence staining images of CD31 (green), and Nile Red (red) in the liver tissues of rats (Scale bar = 100 μm). D) H&E staining image of the major organs 3 days after intravenous administration (Group 1 is normal group, Group 2 is PM@QR/RP-NP group, Scale bar = 200 μm). The number of (E) leukocyte, (F) lymphocyte, (G) neutrophil, (H) red blood cell, (I) blood platelet between the normal and PM@QR/RP-NP group (n = 5). Data presented as mean ± SEM. (For interpretation of the references to color in this figure legend, the reader is referred to the Web version of this article.)

To demonstrate the targeting ability of the PM@QR/RP-NP *in vivo*, nile red (NR), nanoparticles labeled with nile red (NR-NP) and PM@NR-NP were injected into IRI rats. Then, the livers and other organs were removed for *ex vivo* 6 h, 12 h, and 24 h after liver IRI. The *ex vivo* images (Fig. 4A) revealed that the fluorescence of the livers in the NR and NR-NP groups gradually increased over time and were significantly stronger at 24 h. However, the liver fluorescence intensity in the PM@NR-NP group was significantly greater than those in the NR and NR-NP groups at 24 h, indicating that the biomimetic nanoparticles increased drug accumulation in the liver. Significant liver tissue accumulation of the PM@NR-NP was observed at different time points after administration, indicating that the PM coating increased targeting to the IRI site in the liver. Furthermore, the distribution of the PM@NR-NP in the liver was greater than that of the NR-NP (Fig. 4B). These results further supported the long circulation time of the PM@QR/RP-NP *in vivo* and their ability to target the IRI regions of the liver. Immunofluorescence staining of drug distribution in rat liver tissues with CD31-labeled vascular endothelial cells (Fig. 4C) showed that PM@NR-NP co-localized better with vascular endothelial cells than NR and NR-NP, suggesting that indicated that the targeting effect of platelet membrane coating improved the uptake of nanoparticles by tissue cells. More importantly, to evaluate the safety of PM@QR/RP-NP, different formulations were injected into rats via the tail vein at a dose of 10 mg kg^−1^, and the major organs were collected 24 h later, made into sections, and stained with hematoxylin and eosin (H&E). The results (Fig. 4D) showed that the livers, lungs, kidneys, and spleens of the rats administered PM@QR/RP-NP displayed no signs of tissue damage or disorder and displayed normal physiological structures and cell morphologies. Moreover, there was no significant difference in the routine indicators evaluated between the rats injected with PM@QR/RP-NP and the normal rats (Fig. 4E–I). These results indicated that the PM@QR/RP-NP were mainly distributed in the liver and had excellent biological safety.

### 3.6 The PM@QR/RP-NP alleviate oxidative stress and modulate macrophage polarization in IRI rats

Although the exact cause of biliary injury after transplantation has not been identified, increasing evidence suggests that excessive oxidative stress in the early stage caused and advanced inflammatory responses mediated by IRI play critical roles. The PM@QR/RP-NP showed excellent abilities in terms of resisting oxidative stress and regulating the macrophage phenotype *in vitro*. Therefore, an IRI rat model was used to investigate whether the PM@QR/RP-NP could alleviate the progression of IRI *in vivo*.

**Fig. 5:**
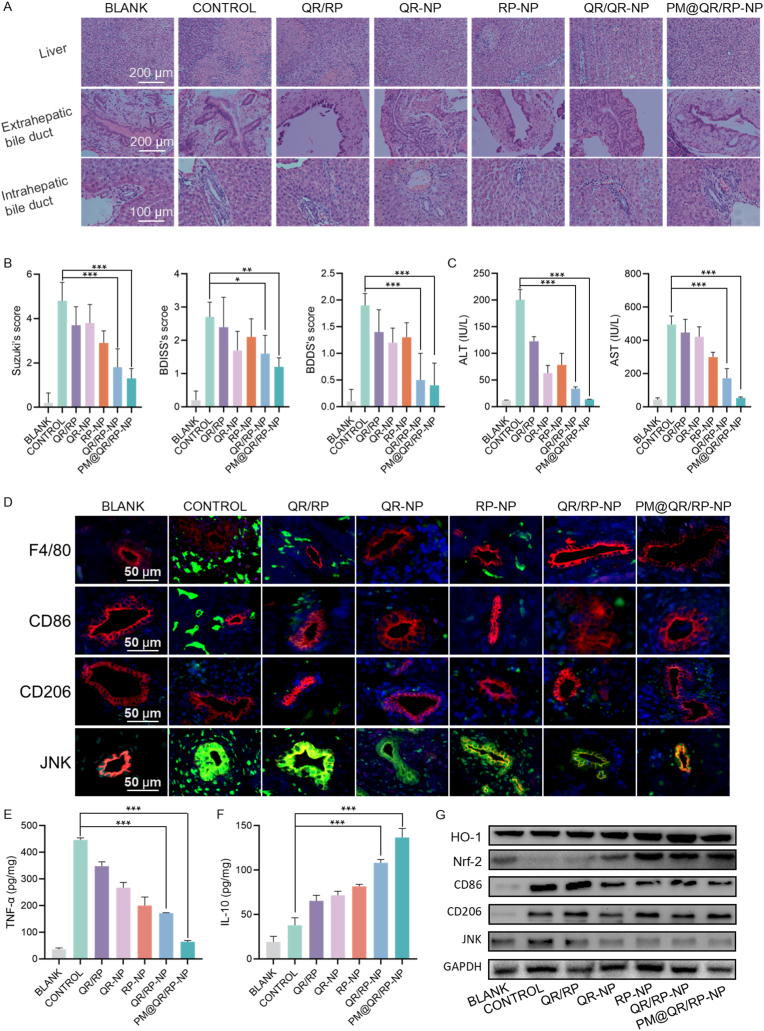
Alleviation of oxidative stress and modulating macrophage polarization in the PM@QR/RP-NP-treated IRI rats. A) H&E staining images of liver tissue (Scale bar = 200 μm), extrahepatic bile duct (Scale bar = 200 μm) duct and intrahepatic bile (Scale bar = 100 μm). B) Suzuki, BDISS and BDDS score of liver, intrahepatic bile duct and Extrahepatic bile duct injury (n = 5). C) Effects of various treatments on serum ALT and AST levels (n = 3). D) Immunofluorescence staining images of F4/80 (green), CD86 (green), CD206 (green), JNK (green), and CK7 (red) in the intrahepatic bile duct tissues of IRI and healthy rats after different treatment (Scale bar = 50 μm). Changes in (E) TNF-α and (F) IL-10 levels in the liver tissues of IRI and healthy rats after different treatments, respectively (n = 5). G) WB analysis showing the expression of antioxidation-related and modulating macrophage polarization proteins in IRI rats after various treatments. Data presented as mean ± SEM. ∗p < 0.05, ∗∗p < 0.01, ∗∗∗p < 0.001. (For interpretation of the references to color in this figure legend, the reader is referred to the Web version of this article.)

The H&E stain images (Fig. 5A) showed that the control rats exhibited obvious tissue damage, including hepatocyte necrosis, biliary epithelial cell polarity disruption, cell detachment, and some localized necrosis. In the QR/RP-NP group, the area of liver tissue necrosis was significantly reduced, cholangiocyte edema was alleviated, and the integrity of biliary tissue was good. The therapeutic effects of the drugs were also evaluated by pathological scoring (Fig. 5B). The Suzuki score, which reflects the degree of hepatic tissue injury, was 4.8 in the control group, and the scores in the QR-NP, RP-NP and QR/RP-NP groups were 3.8, 2.9 and 1.8, respectively, indicating that dual drug therapy could enhance the therapeutic effect of the drugs *in vivo*. The BDISS and BDDS were calculated as a reflection of the extent of intrahepatic and extrahepatic biliary injury, respectively [49,50]. These scores were 1.2 and 1.7 in the QR-NP group, 1.3 and 2.1 in the RP-NP group, and 0.5 and 1.6 in the QR/RP-NP group, respectively, showing a significant difference. These data indicated that dual drug therapy enhanced the therapeutic effect each drug on biliary injury *in vivo*.

Furthermore, the levels of ALT and AST (Fig. 5C) were 200.5 IU L^−1^ and 495.2 IU L^−1^ in the control group, but decreased by 6.83 % (to 13.7 IU L^−1^) and 11.81 % (to 52.9 IU L^−1^) in the PM@QR/RP-NP group, respectively. These changes in marker levels were consistent with the pathological scores and collectively suggest that PM@QR/RP-NP treatment significantly alleviated the pathological progression of IRI.

PM@QR/RP-NP reduced oxidative stress and regulate the phenotypic polarization of RAW264.7 cells *in vitro*. Thus, whether the PM@QR/RP-NP had similar effects *in vivo* was further explored. Immunofluorescence staining (Fig. 5D) was used to evaluate the expression of M1 (CD86) and M2 (CD206) macrophage markers in the tissues surrounding biliary epithelial cells. Compared with that in the normal group, the expression of the M1 marker increased in the IRI group, while the expression of the M2 marker decreased, and these changes were reversed after PM@QR/RP-NP treatment. In addition, we determined the levels of the cytokines TNF-α and IL-10 produced by M1/2 macrophages in the serum via ELISA (Fig. 5E and F). Notably, the serum level of TNF-α was 446.58 pg mg^−1^ in the control group, which was significantly reduced to 64.03 pg mg^−1^ in the PM@QR/RP-NP group along with an increase in the level of anti-inflammatory cytokines, which indicated that PM@QR/RP-NP could reduce the inflammatory response. The WB results (Fig. 5G) showed that the protein expression levels of Nrf-2 and HO-1 were significantly elevated after PM@QR/RP-NP treatment, suggesting that PM@QR/RP-NP enhanced the antioxidative stress capacity *in vivo*. It also corroborated to demonstrate that PM@QR/RP-NP significantly reduced the expression of CD86 and JNK signaling pathways, while increasing the expression of CD206. Therefore, the PM@QR/RP-NP significantly alleviated biliary injury caused by IRI, which may be related to QR mitigating oxidative stress-induced damage by increasing Nrf-2/HO-1 pathway activation in the early stage and RP modulating macrophage polarization to suppress the JNK pathway in the advanced stage.

### 3.7 The PM@QR/RP-NP alleviate oxidative stress and modulate macrophage polarization in liver transplantation rats

The results of the IRI model suggested that the PM@QR/RP-NP attenuated oxidative stress and modulated macrophage polarization. Next, PM@QR/RP-NP were injected into donor rats before transplantation and recipient rats every other day after transplantation via the tail vein. Tissue samples were collected on the 1st and 7th day after liver transplantation to further investigate the ability of the nanoparticles to mitigate biliary injury.

**Fig. 6:**
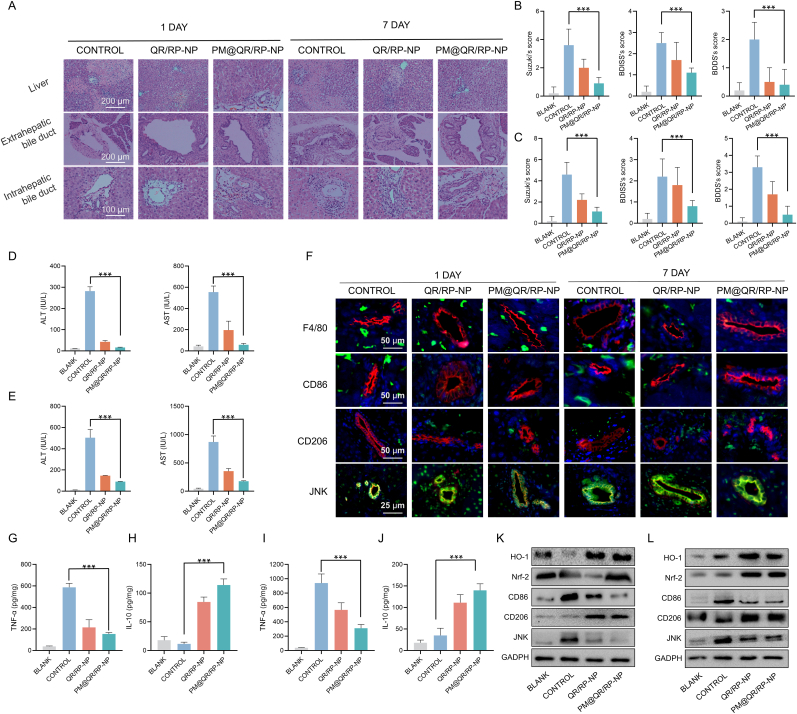
Alleviation of oxidative stress and modulating macrophage polarization in the PM@QR/RP-NP-treated liver transplantation rat. A) Liver tissue (Scale bar = 200 μm), extrahepatic bile duct (Scale bar = 200 μm) and intrahepatic bile duct (Scale bar = 100 μm) analysis using H&E staining in each group on the 1st and 7th day after liver transplantation. Suzuki, BDISS and BDDS score of liver, intrahepatic bile duct and extrahepatic bile duct injury on the (B) 1st and (C) 7th day after liver transplantation (n = 5). Effects of various treatments on serum ALT and AST levels on the (D) 1st and (E) 7th day (n = 3). F) Immunofluorescence staining images of F4/80 (green), CD86 (green), CD206 (green), JNK (green) and CK7 (red) in the intrahepatic bile duct tissues of liver transplantation rats after different treatment (Scale bar = 50 μm). (G) TNF-α and (H) IL-10 levels in the liver tissues of liver transplantation and healthy rats after different treatments on the 1st day (n = 5). (I) TNF-α and (J) IL-10 levels in the liver tissues of liver transplantation and healthy rats after different treatments on the 7th day (n = 5). WB analysis showing the expression of antioxidation-related and modulating macrophage polarization proteins in liver transplantation rats after various treatments on the (K) 1st and (L) 7th day. Data presented as mean ± SEM. ∗p < 0.05, ∗∗p < 0.01, ∗∗∗p < 0.001. (For interpretation of the references to color in this figure legend, the reader is referred to the Web version of this article.)

H&E staining (Fig. 6A) revealed extensive tissue damage in the liver transplantation model rats, with significantly more liver tissue necrosis on the 7th day than on the 1st day; the biliary tissue was also clearly irregular and disrupted. Notably, the PM@QR/RP-NP exhibited good therapeutic effects on both the 1st day and the 7th day posttransplantation, as inflammatory cell infiltration in the liver tissue was reduced, bile duct continuity was restored, and the bile duct lumen became regular. The Suzuki, BDISS and BDSS scores of the control group were 3.2, 2.42 and 2.0 and 4.1, 3.08 and 2.12 on days 1 and 7, respectively (Fig. 6B and C), indicating that the extents of tissue damage and biliary injury were significantly greater on the 7th day after transplantation than on the 1st day. However, the PM@QR/RP-NP treatment group had significantly lower pathological scores on days 1 and 7 (0.88, 0.33, and 1.10 and 1.25, 0.41 and 0.65, respectively) and consistent H&E staining results. These data suggested that PM@QR/RP-NP treatment significantly reduced biliary histopathological damage. To directly confirm the protective effect on bile duct cells, indicators of cholangiocyte injury and regeneration were examined. CK19 [51], N-cadherin [52], and EpCAM [53] are reflective of the intensity of the bile duct response, key markers of epithelial-mesenchymal transition (EMT), and markers of regenerating epithelial cells, respectively. The results of the WB experiments (Fig. S5 and 6) showed that hepatic ischemia/reperfusion injury resulted in bile duct reactive hyperplasia (bile duct response) and EMT, and the expressions of CK19 and N-cadherin were significantly elevated. Compared with the control and QR/RP-NP groups, PM@QR/RP-NP treatment enhanced the regeneration of cholangiocytes with a significant increase in the expression of EpCAM and a decrease in the expression of CK19 and N-cadherin, which further demonstrated that PM@QR/RP-NP was effective in promoting the regeneration of cholangiocytes through drug treatment. In the control group, on the 1st day after surgery, the ALT and AST levels were 282.2 IU L^−1^ and 554.6 IU L^−1^, respectively, but after treatment with PM@QR/RP-NP, these values were reduced to 5.73 % (16.17 IU L^−1^) and 10.38 % (57.57 IU L^−1^) of their original levels, respectively. Furthermore, on the 7th day, the levels of ALT and AST in the control group were 504.6 IU L^−1^ and 868.8 IU L^−1^, respectively, and after treatment with PM@QR/RP-NP, these values were reduced to 17.94 % (90.53 IU L^−1^) and 20.21 % (175.6 IU L^−1^) of their original levels, respectively. These findings further indicate that the damage that occurs in the advanced-stage after transplantation can worsen and further affect biliary function, whereas PM@QR/RP-NP treatment can alleviate these effects.

Given that biliary injury after liver transplantation has been attributed mainly to oxidative stress in the early stage and macrophage polarization-mediated inflammatory injury in the advanced stage, the expression of macrophage markers and inflammation-related factors in intrahepatic bile duct tissues was further assessed via immunofluorescence staining (Fig. 6F). In the control group, the strong fluorescence signal of CD86 around the cholangiocytes, the weak signal of CD206 and the strong intensity of JNK on the 1st and 7th day after transplantation were consistent with the previous pathological results, indicating that the damage on the 7th day after liver transplantation was more severe than that on the 1st and suggesting more complex damage in the later stage. In the PM@QR/RP-NP group, the fluorescence signal of JNK and CD86 was weakened with the fluorescence intensities of CD206 were enhanced, indicating that PM@QR/RP-NP treatment may alleviate biliary injury by regulating macrophage polarization.

In addition, the serum levels of the cytokines TNF-α and IL-10 were analyzed via ELISA (Fig. 6G–J). The results revealed that the levels of TNF-α in the control group were 587.40 pg mg^−1^ and 970.60 pg mg^−1^ on the 1st and 7th day, respectively, suggesting that more inflammatory factors were produced by M1-polarized macrophages in the advanced stage. PM@QR/RP-NP significantly decreased the level of TNF-α by 26.07 % (to 153.16 pg mg^−1^) and 31.95 % (to 310.14 pg mg^−1^) while increasing the level of IL-10, suggesting that these nanoparticles can inhibit inflammatory responses *in vivo*. These results suggested that PM@QR/RP-NP could alleviate inflammation during liver transplantation by participating in the regulation of macrophage polarization. The significant increase in the gray values of the Nrf-2 and HO-1 bands in the treatment group (Fig. 6F and G) indicated that oxidative stress was mitigated after drug treatment. The expression of CD86 and JNK were decreased, whereas the expression of CD206 was increased after treatments, suggesting that the PM@QR/RP-NP promoted the polarization of M1-type macrophages to M2-type macrophages, thereby alleviating biliary injury induced by inflammation. Taken together, these results suggest that PM@QR/RP-NP might inhibit the damage caused by oxidative stress and macrophage polarization induced by IRI after liver transplantation *in vivo*.

## 4 Conclusion

In summary, we prepared platelet-coated biomimetic nanoparticles loaded with the drugs QR and RP (PM@QR/RP-NP) via coprecipitation to reduce biliary injury caused by IRI after liver transplantation. The PM enhanced targeting to damaged vascular ECs and macrophages. In IRI and liver transplantation rat models, we confirmed that the PM@QR/RP-NP released QR effectively in the early stage of IRI to activate Nrf-2/HO-1 signaling and reduce oxidative stress injury, while in the advanced stage, RP regulated macrophage polarization to reduce inflammation by suppressing JNK signaling. Thus, biliary injury was inhibited after liver transplantation by synergistically treating both the early and advanced stages. Overall, these biomimetic nanoparticles have great potential for treating biliary injury after liver transplantation.

## CRediT authorship contribution statement

**Tian Dong:** Writing – original draft, Project administration, Methodology, Investigation, Formal analysis, Conceptualization. **Chengcheng Zhang:** Writing – review & editing, Project administration, Investigation, Formal analysis. **Zhaoyi Wu:** Project administration, Methodology, Investigation. **Ling Shuai:** Project administration, Methodology, Investigation. **Nengsheng Fu:** Methodology. **Yujun Zhang:** Writing – review & editing, Visualization, Supervision, Resources, Project administration, Methodology, Conceptualization. **Leida Zhang:** Writing – review & editing, Visualization, Validation, Supervision, Resources, Project administration, Methodology, Investigation, Funding acquisition, Conceptualization. **Xiang Xiong:** Writing – review & editing, Writing – original draft, Visualization, Validation, Methodology, Investigation, Funding acquisition.

## Declaration of competing interest

The authors declare no conflict of interest.

## Data Availability

Data will be made available on request.
