# Supplementary material for: A biomimetic nanomedicine alleviates liver transplant-related biliary injury by sequentially inhibiting oxidative stress and regulating macrophage polarization via Nrf-2/HO-1 and JNK pathways
Source: Mater Today Bio. 2025 Apr 23;32:101797. doi: 10.1016/j.mtbio.2025.101797 (PMC12059350; doi:10.1016/j.mtbio.2025.101797)
Supplement: Multimedia component 1 [file mmc1.docx]

**Supporting Information**

**A Biomimetic Nanomedicine Alleviates Liver Transplant-Related Biliary Injury by Sequentially Inhibiting Oxidative Stress and Regulating Macrophage Polarization via Nrf-2/HO-1 and JNK Pathways**

Tian Dong ^1, #^, Chengcheng Zhang^1, #^, Zhaoyi Wu ^1^, Ling Shuai ^1^, Nengsheng Fu ^1^, Yujun Zhang ^1,^ *, Leida Zhang ^1,^ *, and Xiang Xiong ^1,^ *

^1^ Key Laboratory of Hepatobiliary and Pancreatic Surgery, Institute of Hepatobiliary Surgery, Southwest Hospital, Third Military Medical University (Army Medical University), Chongqing 400038, China

^*^ Correspondence:

Yujun Zhang, zhangyu55583@tmmu.edu.cn; Leida Zhang, [zldxngd@163.com](mailto:zldxngd@163.com); Xiang Xiong, xiongxiang@tmmu.edu.cn

^#^ These authors contributed equally to this work.

**Materials**

Total Antioxidant Capacity Assay Kit (S0119), CCK-8 Kit (C0038), Reactive Oxygen Species Assay Kit (S0033M), Mitochondrial Membrane Potential Assay Kit (C2006), TUNEL Apoptosis Detection Kit (C1086), Lysosomal Green Fluorescent Probe (C1047S), Apoptosis Detection Kit (C1062S), HO-1/Heme Oxygenase 1 Rabbit Monoclonal Antibody (AG2181) were purchased from Shanghai Biyuntian Biotechnology Co (Shanghai, China). Superoxide Dismutase Activity Assay Kit (BC0175) was purchased from Beijing Solepol Technology Co (Beijing, China). Mouse TNF-α ELISA Kit (EM0183), Mouse IL-10 ELISA Kit (EM0100), Rat IL-10 ELISA Kit, Rat TNF-α ELISA Kit (ER1393) were purchased from Fine Biotech. Brilliant Violet 421™ anti-mouse CD206 (MMR) Antibody (141717), PE anti-mouse CD86 Antibody (105007) were purchased from BIOLEGEND (Beijing) Biotechnology Co (Beijing, China). Polyclonal Antibody (AF07082), JNK1/2/3 (phospho Thr183) Polyclonal Antibody (AF00639), F4/80 Rabbit pAb (SAF002), CK19 (AF10701) were purchased from Hunan Aifang Biotechnology Co (Wuhan, China). CD86 (C- terminal) Polyclonal antibody (26007) was purchased from BIOLEGEND (Beijing) Biotechnology Co. terminal) Polyclonal antibody (26903-1-AP), NRF2, NFE2L2 Polyclonal antibody (16396-1-AP), GAPDH Monoclonal antibody (60004-1-Ig) were purchased from Wuhan Three Eagles Bio-technology Co. Ltd (Wuhan, China). EPCAM (ab213500), N-cadherin (CDH2) (ab18203) were purchased from Abcor (Shanghai, China) Trading Co.

Cell lines and animals. Human umbilical vein endothelial cell line HUVEC was obtained from Southwest Jiaotong University (Chengdu, China), mouse monocyte macrophage leukemia cells (RAW264.7) were obtained from Third Military Medical University (Chongqing, China), rat extrahepatic bile duct epithelial cells (RAT-iCell-d016) were purchased from Cellverse Bioscience Technology Co., Ltd (Shanghai, China). Cells were cultured in dulbecco's modified eagle medium (DMEM) high glucose medium (Hyclone), containing 10% fetal bovine serum (Bioind) at 37°C in a 5% CO2 humidified environment incubator (Thermo Scientific, USA).

All in vivo animal-related experiments were approved by the Institutional Animal Care and Use Committee (IACUC) of Army Medical University and complied with all relevant ethical regulations (AMUWEC20242047). SD rats were maintained in a temperature-controlled, specific-pathogen-free (SPF) animal laboratory in the Animal Center of Army Medical University.

.
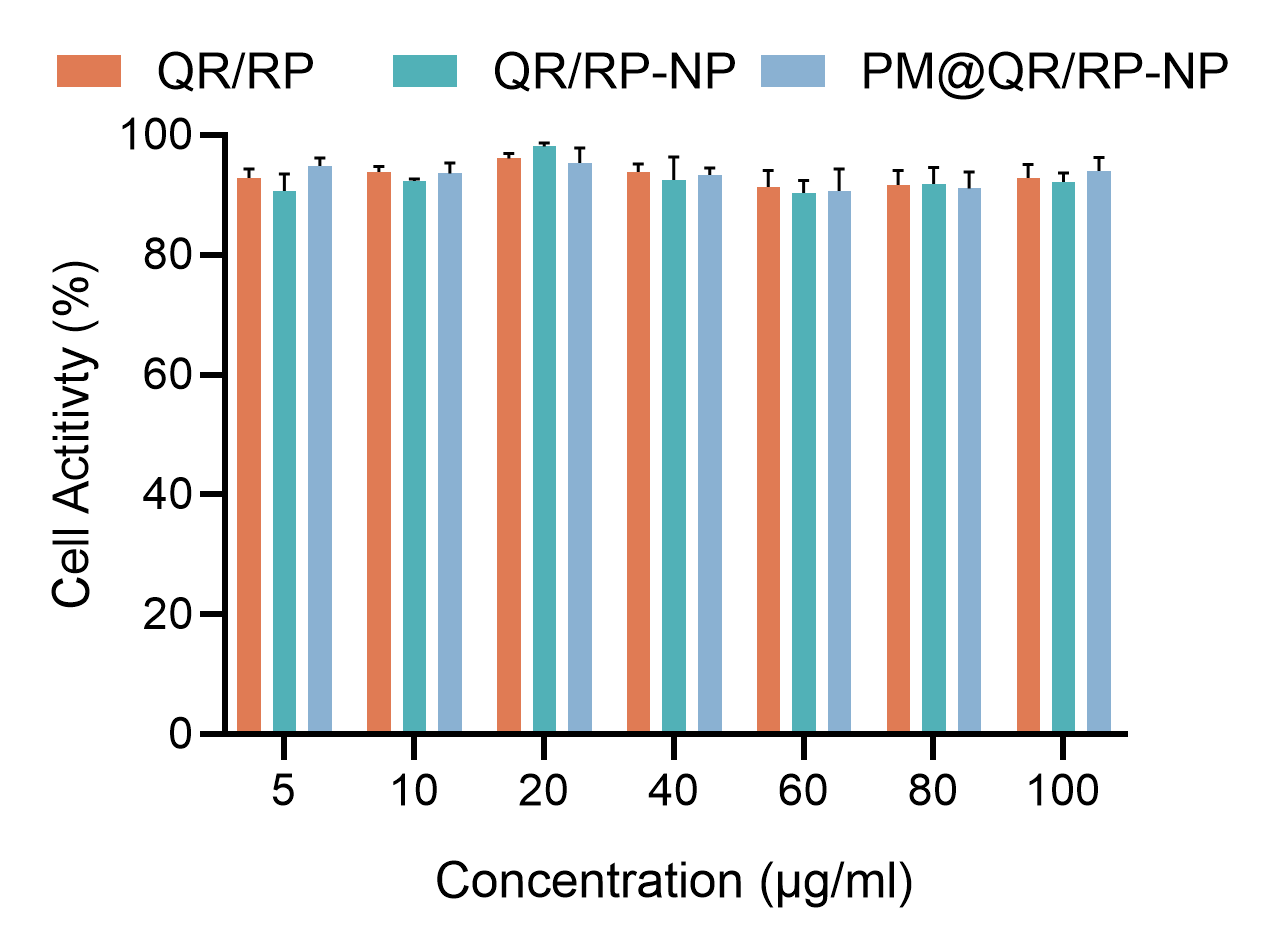


Figure. S1 Effects of nanoparticle on ECs viability evaluated by CCK-8 assay.


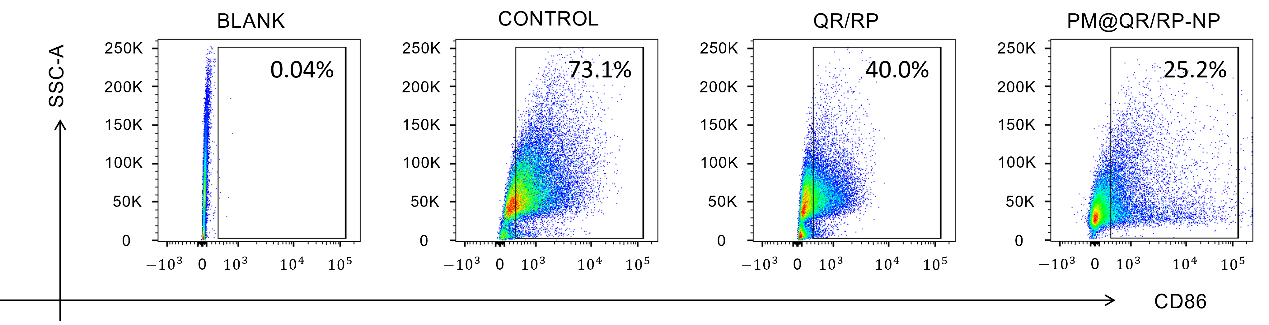


Figure S2. Flow results of the influence of reactive oxygen species on the polarization of RAW264.7 stimulated by ECs with different treatments.


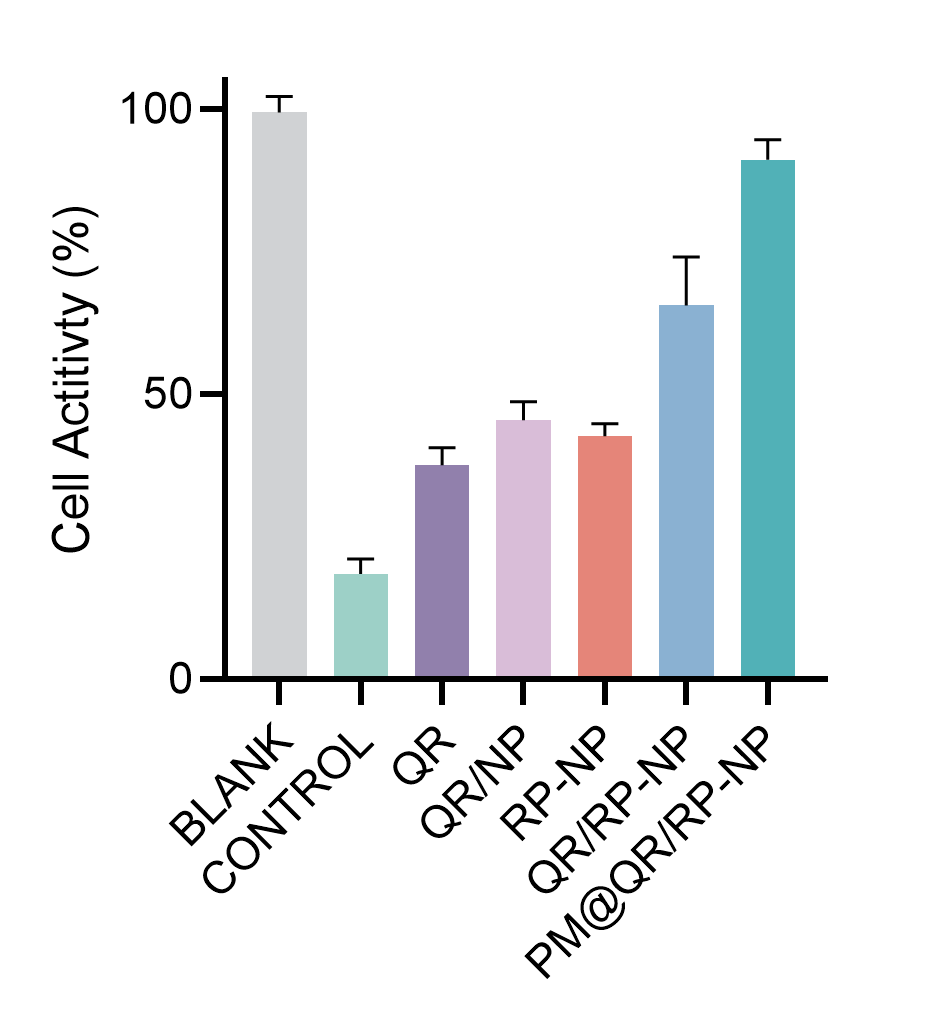


Figure S3. CCK-8 results of the effect of RAW264.7 polarization on extrahepatic bile duct epithelial cells.


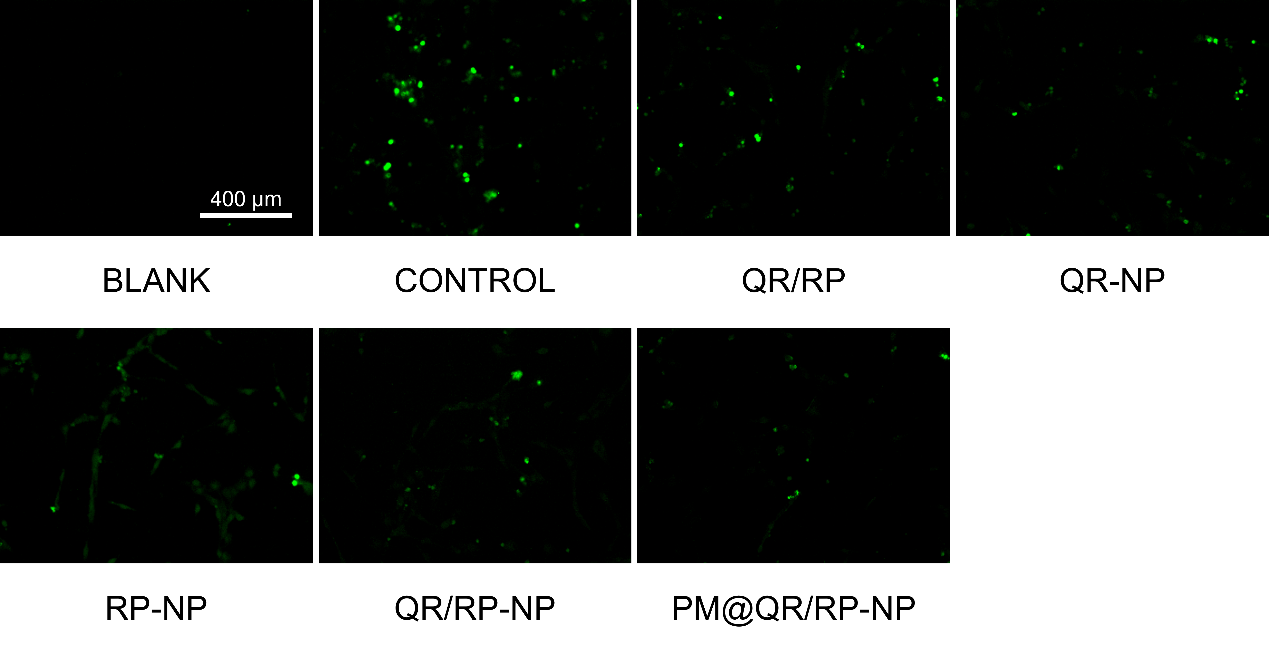


Figure S4. TUNEL staining of the effect of RAW264.7 polarization on extrahepatic bile duct epithelial cells.


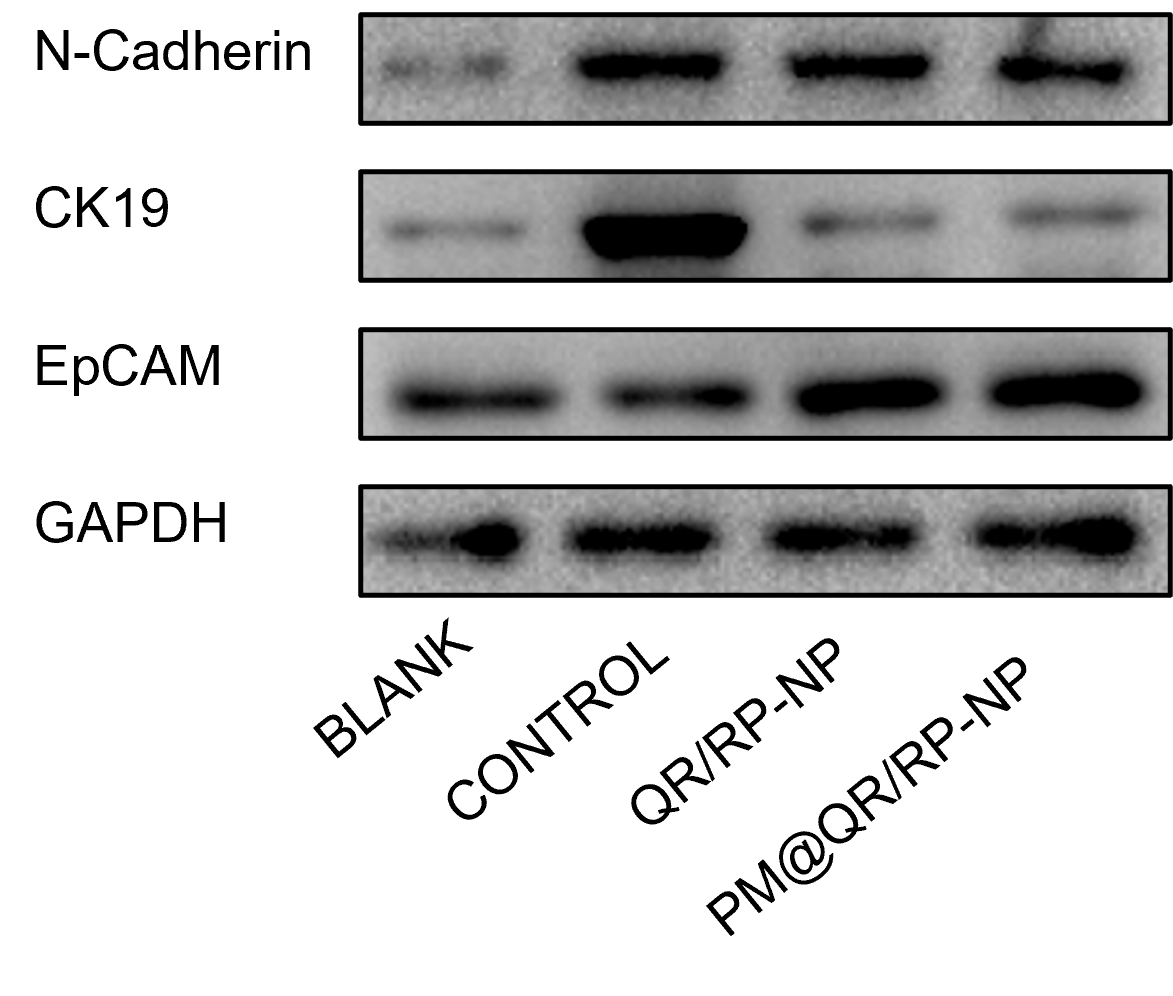


Fig S5. WB analysis showing the expression of antioxidation-related and modulating macrophage polarization proteins in liver transplantation rats after various treatments on the 1st day


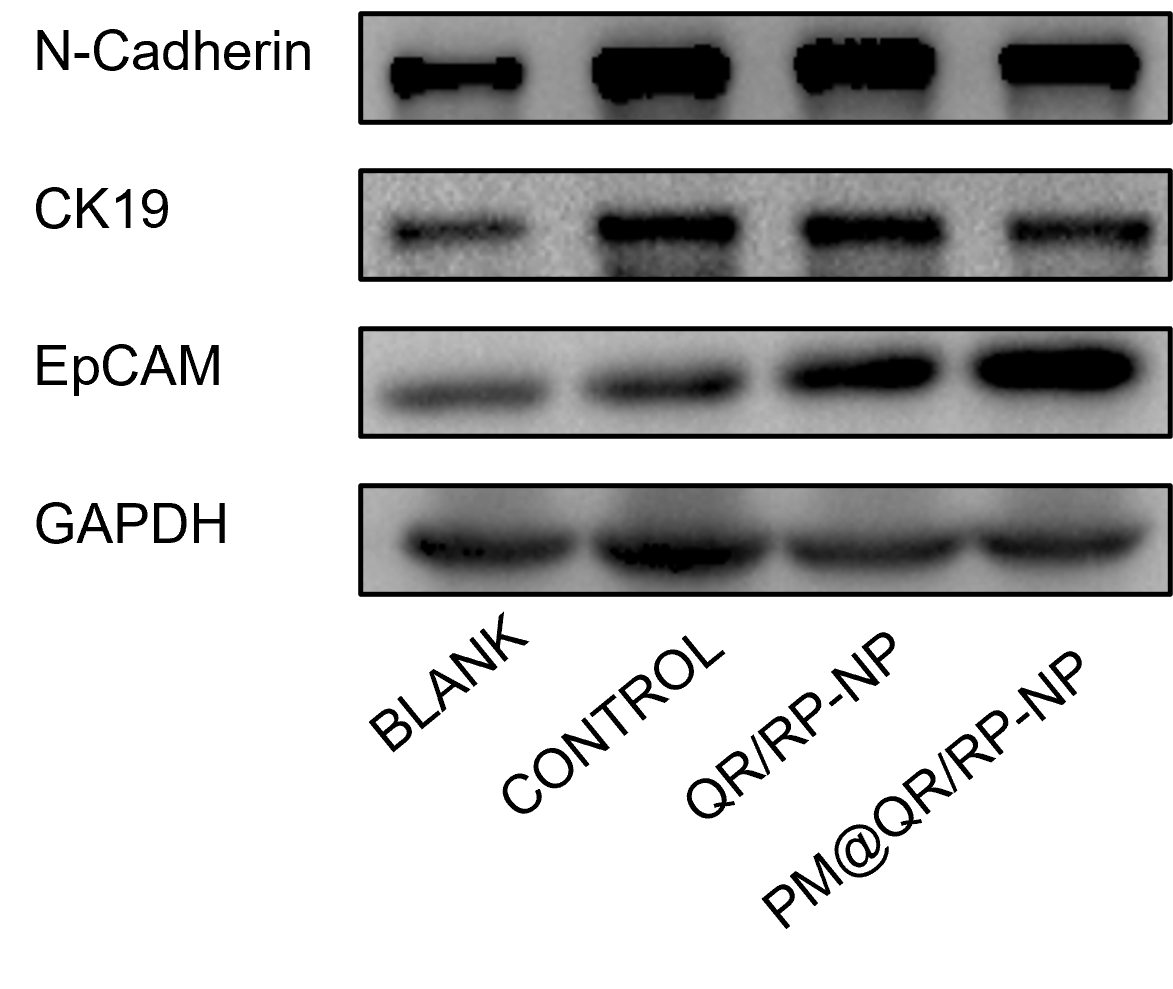


Fig S6. WB analysis showing the expression of antioxidation-related and modulating macrophage polarization proteins in liver transplantation rats after various treatments on the 7th day

Table S1. LC and LE of QR and RP in PM@QR/RP-NP

|  | LC (%) | LE (%) |
| --- | --- | --- |
| QR in PM@QR/RP-NP | 3.14 ± 0.65% | 61.38 ± 4.64% |
| RP in PM@QR/RP-NP | 1.02 ± 0.45% | 43.55 ± 6.21% |
